# Supplementary material for: The Individual Effects of Cyclin-Dependent Kinase Inhibitors on Head and Neck Cancer Cells—A Systematic Analysis
Source: Cancers (Basel). 2021 May 15;13(10):2396. doi: 10.3390/cancers13102396 (PMC8157193; doi:10.3390/cancers13102396)
Supplement: Supplementary file 1 [file cancers-13-02396-s001.zip › cancers-1148175-SI.pdf]

The Individual Effects of Cyclin-Dependent Kinase Inhibitors on Head and Neck Cancer Cells—A Systematic Analysis

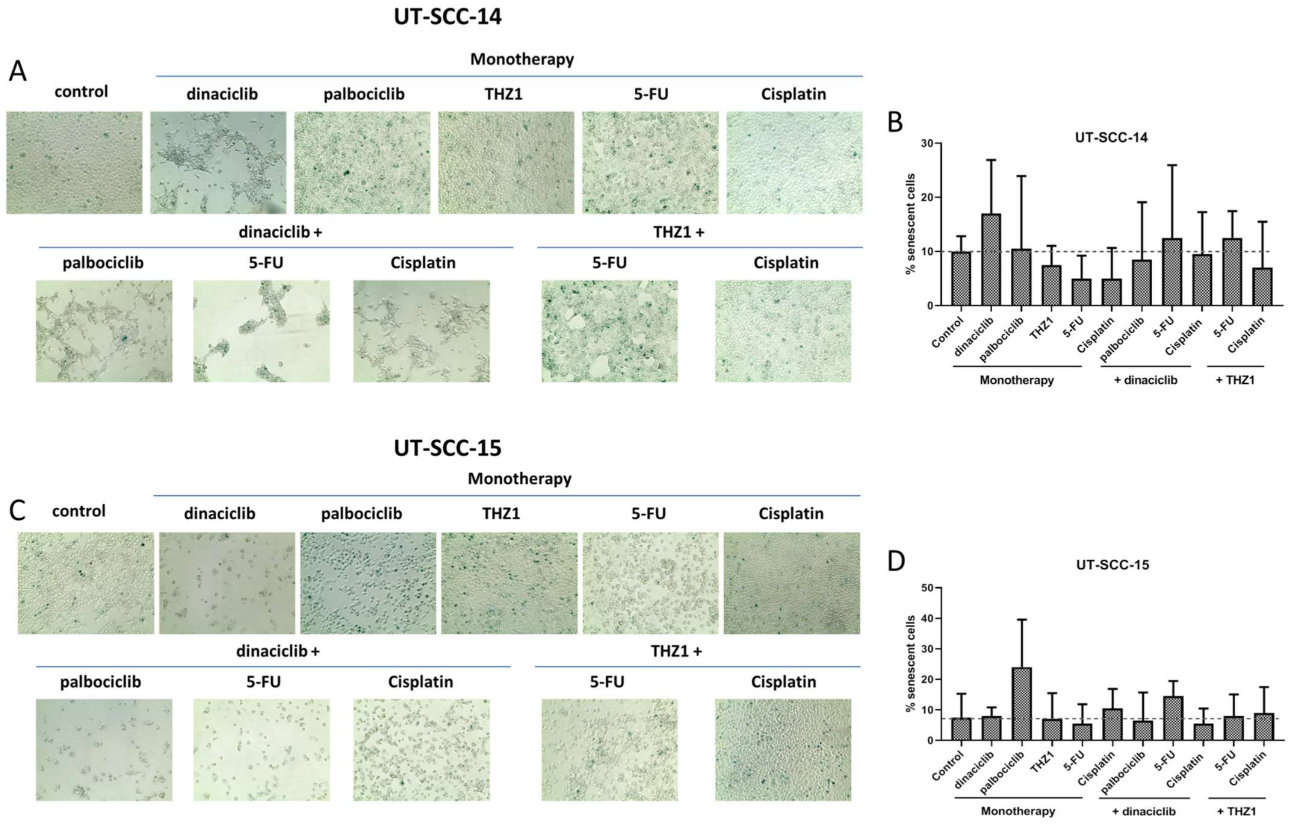

Figure S1: Senescence. (A,B) UT-SCC-14, and (C,D) UT-SCC-15 cells. Cells were treated with test substances for 72 h, fixed and stained with β-galactosidase staining solution overnight at 37 °C without CO_2. The blue spots show ß-galactosidase-activity, indicative for senescence. Representative light microscopy images were shown in (A) UT-SCC-14 and (C) UT-SCC-15. In (B) UT-SCC-14 and (D) UT-SCC-15 quantitative analyses of senescent cells in relation to whole cell were shown. Drug doses were as follows: dinaciclib [0.02 µM]; palbociclib [1 µM]; THZ1 [UT-SCC14: 0.02 µM; UT-SCC-15: 0.005 µM]; 5-FU [90 µg/ml]; Cisplatin [0.1 µg/ml].

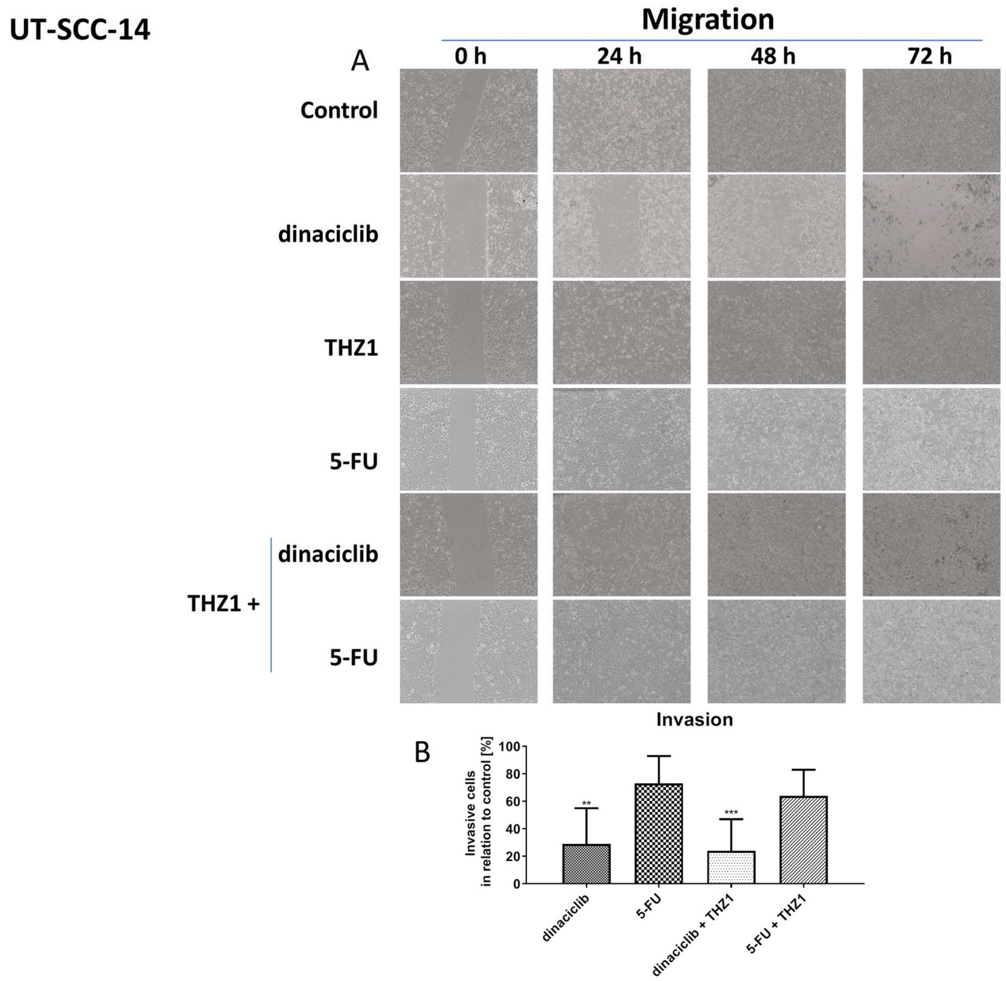

Figure S2: Scratch-Assay and Invasiveness UT-SCC-14. Cells were seeded and grown to confluence. Then a scratch was placed on the plate utilizing a 200 µl pipette tip. Detached cells were removed, and test substances were added. The scratch was monitored for 3 days by light microscopy until the scratch was closed or most cells die due to the cytotoxic effects of the test substances. Drug doses were as follows: dinaciclib [0.02 µM]; THZ1 [0.02 µM]; 5-FU [90 µg/ml]. For invasion assay, inserts were coated with Matrigel and cells seeded in serum free, treatment containing media. The inserts were placed into a 24-well plate containing media with 10 % FCS. Invasiveness was analyzed by WST-1 assay. Displayed are the invasive cells in relation to control after 72 h. Drug doses were as follows: dinaciclib [0.02 µM]; THZ1 [0.02 µM]; 5-FU [90 µg/ml]. 1way ANOVA ($n \geq 4$ independent experiments) ** $p < 0.01$, *** $p < 0.001$ vs. control.

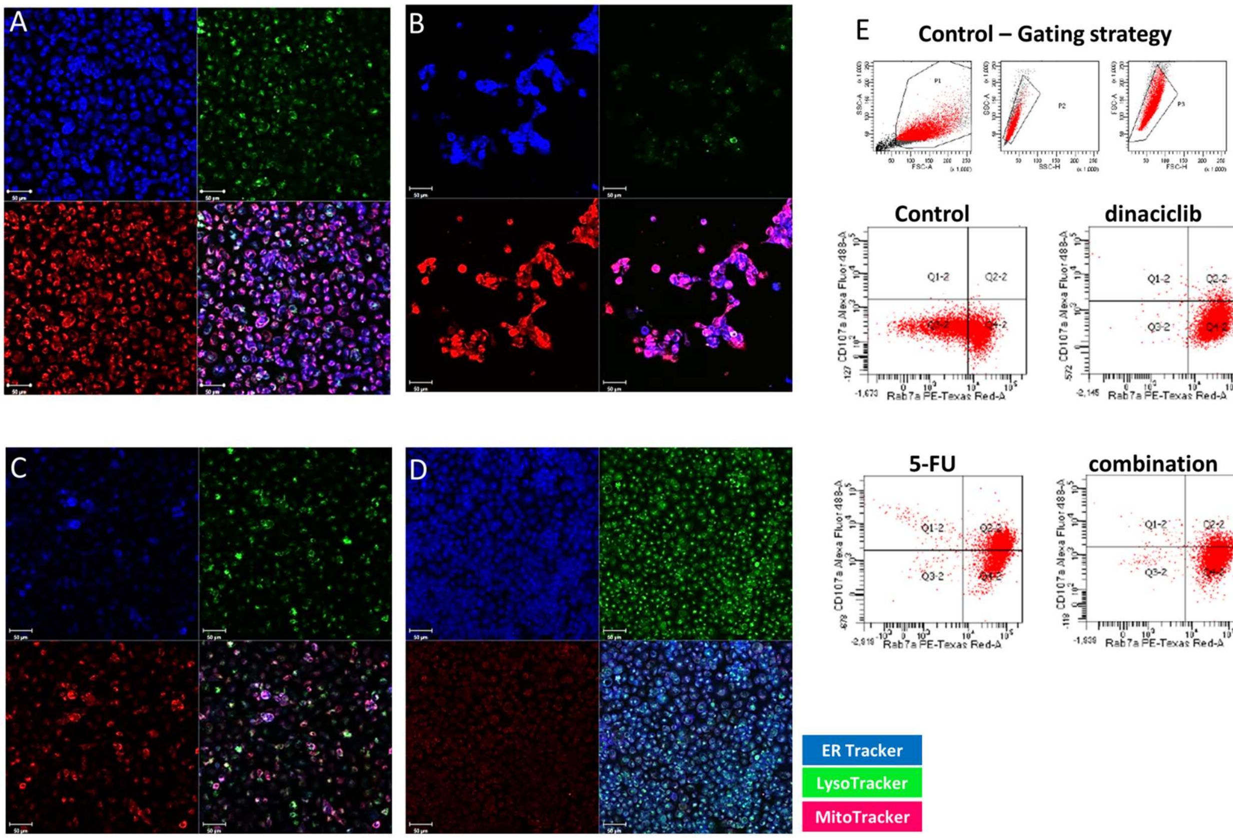

Figure S3: Influence on mitochondria, lysosomes, ER, and vacuole formation. To investigate the effect of the test substances on the mitochondrial activity, the lysosome formation and the ER, cells were treated for 72 h with test substances and stained with Mito-Tracker (red), LysoTracker (green) and ER-Tracker (blue). (A–D) Representative images are shown. (A) positive for all markers (B) positive for ER- and MitoTracker, negative for LysoTracker (C) positive for LysoTracker and MitoTracker, negative for ER-Tracker (D) positive for ER-Tracker and LysoTracker, negative for MitoTracker. Analysis was performed with a ZEISS Elyra 7 Confocal Laser Microscope. (E) Cells were stained for CD107a and Rab7a as a hint for vacuole formation and measured via flow cytometry. Shown is a representative gating strategy and dot plots of selected treatment regimes.

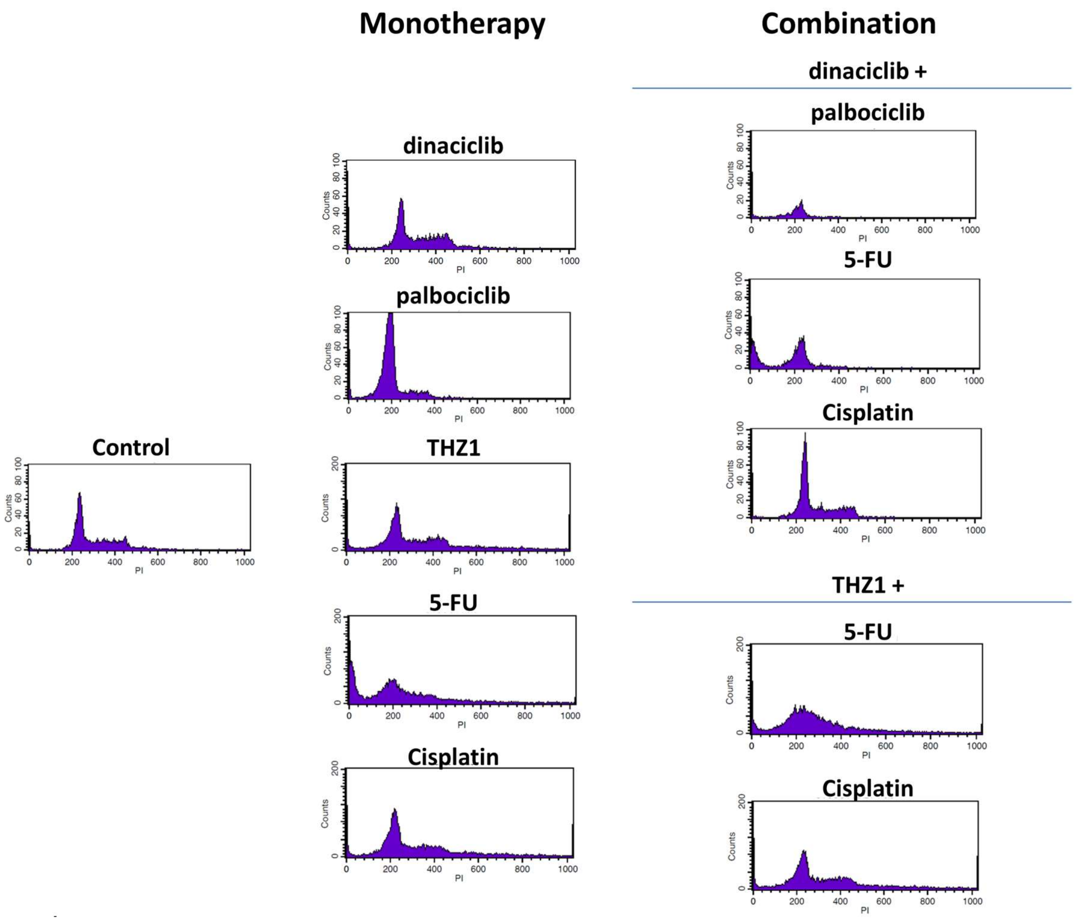

Figure S4: Cell cycle analysis. Ethanol-fixed cells were stained with PI. Representative histograms showing the distribution of individual cell cycle phases (G1, S, G2) in controls and under therapy. Drug doses were as follows: dinaciclib [0.005 µM]; palbociclib [1 µM]; THZ1 [UT-SCC14: 0.02 µM; UT-SCC-15: 0.005 µM]; 5-FU [90 µg/ml]; Cisplatin [0.1 µg/ml].
